# Differences in physical activity domains, guideline adherence, and weight history between metabolically healthy and metabolically abnormal obese adults: a cross-sectional study

**DOI:** 10.1186/s12966-015-0227-z

**Published:** 2015-05-16

**Authors:** Thirumagal Kanagasabai, Niels A. Thakkar, Jennifer L. Kuk, James R. Churilla, Chris I. Ardern

**Affiliations:** School of Kinesiology and Health Science, 352 Norman Bethune College, York University, 4700 Keele Street, Toronto, ON M3J1P3 Canada; Brooks College of Health, University of North Florida, Jacksonville, FL USA

**Keywords:** Physical activity domain, Physical activity guideline adherence, Weight history, Metabolically health obesity, Metabolically abnormal obesity

## Abstract

**Background:**

Despite the accepted health consequences of obesity, emerging research suggests that a significant segment of adults with obesity are metabolically healthy (MHO). To date, MHO individuals have been shown to have higher levels of physical activity (PA), but little is known about the importance of PA domains or the influence of weight history compared to their metabolically abnormal (MAO) counterpart.

**Objective:**

To evaluate the relationship between PA domains, PA guideline adherence, and weight history on MHO.

**Methods:**

Pooled cycles of the National Health and Nutritional Examination Survey (NHANES) 1999–2006 (≥20 y; BMI ≥ 30 kg/m^2^; N = 2,753) and harmonized criteria for metabolic syndrome (MetS) were used. Participants were categorized as “inactive” (no reported PA), “somewhat active” (>0 to < 500 metabolic equivalent (MET) min/week), and “active” (PA guideline adherence, ≥ 500 MET min/week) according to each domain of PA (total, recreational, transportation and household). Logistic and multinomial regressions were modelled for MHO and analyses were adjusted for age, sex, education, ethnicity, income, smoking and alcohol intake.

**Results:**

Compared to MAO, MHO participants were younger, had lower BMI, and were more likely to be classified as active according to their total and recreational PA level. Based on total PA levels, individuals who were active had a 70 % greater likelihood of having the MHO phenotype (OR = 1.70, 95 % CI: 1.19–2.43); however, once stratified by age (20–44 y; 45–59 y; and; ≥60 y), the association remained significant only amongst those aged 45–59 y. Although moderate and vigorous PA were inconsistently related to MHO following adjustment for covariates, losing ≥30 kg in the last 10 y and not gaining ≥10 kg since age 25 y were significant predictors of MHO phenotype for all PA domains, even if adherence to the PA guidelines were not met.

**Conclusion:**

Although PA is associated with MHO, the beneficial effects of PA may be moderated by longer-term changes in weight. Longitudinal analysis of physical activity and weight change trajectories are necessary to isolate the contribution of duration of obesity, PA behaviours, and longer-term outcomes amongst MHO individuals.

## Background

Obesity has manifested into a worldwide epidemic that threatens to shorten life expectancy and the population health of future generations [1]. Recent estimates in the U.S. report that approximately 36 % of the adult population have obesity [2]. The increased health risks due to obesity are well documented, particularly for type II diabetes and cardiovascular disease [3, 4]. The metabolic syndrome (MetS), a cluster of cardiometabolic risk factors which includes central obesity, is associated with a five-fold increased risk of type II diabetes, and a two-fold greater risk of cardiovascular disease [4, 5].

While a generalized obesity phenotype is commonly associated with metabolic dysfunction [3], a significant portion of individuals with obesity appear to be “metabolically healthy” [6]. Using the Harmonized definition of MetS [7] in conjunction with inflammatory markers, Wildman et al. [8] report that 29.2 % of obese men and 35.4 % of obese women in the U.S. are “metabolically healthy obese” (MHO). Because the prevalence of MHO is dependent on the definition used, estimates of MHO have been reported to be as low as 3.3 %, or as high as 43.3 % [6].

Of the many risk factors for metabolic dysfunction, habitual physical activity (PA) may be a particularly potent factor in the prevention and management of MetS and its individual components [9]. Indeed, analyses from the National Health and Nutrition Examination Survey (NHANES) reveal an inverse association between the prevalence of MetS and total PA. In this study, those who met the Department of Health and Human Services (DHHS) guidelines for PA had a 33 % lower odds of MetS [10]. Although the benefits of PA on metabolic health have been well documented in both obese and non-obese populations [11, 12], only a few studies to date have examined the individual domains of PA (e.g., household-, transportation-, and recreation-related PA), in relation to MHO. Given the dominance of health promotion messages regarding fractionalized activity bouts and lifestyle activity [13], a broader understanding of the relationship between each subdomain and metabolic health is necessary.

Moreover, longer-term PA habits are an important predictor of weight history, and longer-term habitual PA, particularly at higher intensity, has been shown to minimize weight gain [14]. Increasing time since weight gain has also been associated with a higher odds of MetS, independent of PA [15], and a recent report suggests that obesity at age 25 is a good predictor of future obesity, cardiometabolic risk, and inflammation [16]. Beyond these findings, the extent to which weight history and PA domains may interact and collectively account for differences in the MHO phenotype is not yet known. Given the documented challenges of effective long-term obesity management [17], exploration of the MHO-PA and MHO-weight history relationships may provide new insight into the appropriateness of weight and activity-based interventions for individuals with obesity. The purpose of this paper is to therefore examine the relationship between PA domains and weight history on the MHO phenotype.

## Methods

### Participants

The NHANES is a comprehensive assessment of health and nutrition in the U.S. population [18]. Each year approximately 5,000 persons are sampled, with data released on a bi-annual basis. The surveying process consists of personal interviews, standardized physical examinations, and collecting laboratory samples to obtain data on participants’ diet and nutrition, socio-demographics, and other health-related measures for all ages and ethnicities. Specifically, trained medical professionals performed physical examinations, and collected and analyzed laboratory samples. Because the definition of household PA changed in the NHANES 2007–2008, this analysis is limited to a combined sample of 1999–2006 cycles. The initial sample included 41,474 individuals from 1999–2006 [1999–00: N = 9,965; 2001–02: N = 11,039; 2003–04: N = 10,122, and; 2005–06: N = 10,348]. Subsequent exclusions were made for age (<20 y: N = 21,163), pregnancy (N = 1,169), (Body Mass Index (BMI) < 30 kg/m^2^ (N = 13,491) and missing MetS components (N = 2,898) leaving a final analytic sample of 2,753 [age 20–85 years; 1999–00: N = 624; 2001–02: N = 625; 2003–04: N = 733, and; 2005–06: N = 771]. No effect of survey year was found, and therefore, data for all analyses were pooled across cycles (i.e. 1999–2006).

### Metabolically healthy obese

The Harmonized Criteria for MetS [7] was used to classify MHO on the basis of ≤ 2 of the following indicators: waist circumference (WC) ≥102 cm (men) and ≥88 cm (women); triglyceride ≥1.69 mmol/L (mM); HDL-cholesterol <1.04 mM (men), <1.29 mM (women); blood pressure (systolic BP ≥130 mmHg or diastolic BP ≥85 mmHg); and fasting plasma glucose ≥5.6 mM. Consequently, metabolically abnormal obese (MAO) would have 3 or more of the above diagnostic criteria, and the number of MetS components [0, 1, 2, 3, 4, 5] would be the sums of the above criteria. Participants with self-reported use of blood pressure, cholesterol, and diabetes medications were classified as having high blood pressure, dyslipidaemia, and high fasting glucose, respectively. BMI was used to classify obesity (≥30 kg/m^2^) on the basis of measured height and weight. Weight history was assessed by self-reported weight 10 y prior and at age 25 y.

### Physical activity domains

As described in Churilla and Fitzhugh [10], PA was assessed by self-report and classified according to intensity and total volume of activity. To calculate metabolic equivalent (MET) min/week for household PA, moderate and vigorous activities the following MET scores were assumed: 4 MET for household and moderate activities and 8 MET for vigorous activities [19]. MET min/week for recreational PA was calculated using the MET score assigned for each activity by NHANES. Transportation PA (walking or biking) was assigned a value of 4 MET, as per NHANES’s suggestion [19]. Activities accumulated in household, transportation and recreational PA domains were then summed to provide a total PA domain. Participants were categorized as “inactive” (no reported physical activity data), “somewhat active” (<500 MET min/week), and “active” (≥500 MET min/week, i.e. meeting current PA guidelines) for each PA domain [19]. Moderate and vigorous intensity recreational PA were also further divided into six categories [10].

### Weight history

Information on weight history was obtained by self-report [“How much did you weigh 10 years ago?”, and “How much did you weigh at age 25?”]. Responses were converted to kg before estimating the differences (i.e., changes in weight) between the self-reported weights and weight at clinic examination. Changes in weight from 10 y ago and at age 25 y were then categorized as lost ≥30 kg, lost <30 kg, gained 0- <10 kg, gained 10- < 20 kg, gained 20- < 30 kg, gained 30- < 40 kg, and gained ≥40 kg.

### Covariates

Demographic (e.g., age, sex, ethnicity, income, and education) and health behavior (e.g. alcohol intake and smoking history) variables were considered as covariates [10]. Smoking history was defined as being a current (smoke cigarettes now), past (smoked ≥100 cigarettes in one’s life but does not smoke now) or non-smoker (smoked <100 cigarettes in one’s life) [16, 20]. Alcohol intake was classified as high (≥3 drinks per day). Educational attainment was categorized as < high school, high school, and college, and income as < $20,000, $20,000-44,999, and ≥ $45,000 [21].

### Statistical analysis

Mean and standard error (SE) for continuous variables, and percent and SE for categorical variables were estimated. Differences in demographic and behavioural characteristics of MHO vs. MAO participants were assessed by independent t-tests and χ^2^ analysis, as appropriate. The relationship between energy expenditure (per 1000 MET min/wk) and the odds of MHO for each PA domain were predicted with logistic regression analyses. Logistic (dichotomous outcomes) and multinomial logit (polychotomous outcomes) regression analyses were subsequently used to estimate the crude, age, and multivariable (i.e., age, sex, ethnicity, income, education, alcohol intake and smoking history) adjusted odds ratios (OR) and 95 % confidence intervals (CI) for the relationship between PA domains, guideline adherence, and intensity, and the MHO phenotype. The moderating effect of age was explored, and age-stratified analyses were reported. To explore the effect of weight history on MHO phenotype, these analyses were then repeated using weight change from 10 y ago and weight at age 25 y as the primary exposure. For the 10 y weight change analysis, participants under age 30 y were excluded, since they would have been children or adolescents. All analyses were weighted with the MEC examination weight from the demographics data file to be representative of the U.S. population using SAS v9.3 (Cary, NC, U.S.A). Statistical significance was set at an α of 0.05.

## Results

Table 1 presents the descriptive characteristics of MHO and MAO participants. Approximately one third of the obese sample was metabolically healthy (31.6 % (SE: 1.2 %)). In general, MHO individuals were more likely to be younger, women, and non-Hispanic black or Mexican American compared to MAO. Individuals with MHO also had 1.7 kg/m^2^ lower BMI, higher income, higher prevalence of never smoking, and were more likely to be high alcohol consumers than MAO. Individuals who were MHO also engaged in higher levels of total and recreational but not household or transportation PA. In our sample, few obese individuals had zero MetS components (N = 16), while approximately 8 % of the participants (n = 215) had elevated WC without any of the other MetS criteria (data not shown).

**Table 1:** Characteristics of the U.S. adult obese population ≥20 years of age

Characteristics		Metabolically healthy obese (n = 816)	Metabolically abnormal obese (n = 1,937)	*P* value
BMI (kg/m^2^)		34.5 (0.2)	36.2 (0.2)	<0.05
Age (years)		41.6 (0.7)	51.9 (0.5)	<0.05
Age categories				
	<45 years	64.5 (2.6)	33.5 (1.6)	<0.05
	45-59 years	24.0 (2.5)	34.3 (1.4)	
	≥60 years	11.5 (1.3)	32.1 (1.6)	
Sex	Men	40.9 (1.9)	49.9 (1.2)	<0.05
	Women	59.1 (1.9)	50.8 (1.2)	
Ethnicity				
	Non-Hispanic White	63.6 (2.7)	73.8 (1.6)	<0.05
	Non-Hispanic Black	19.1 (1.9)	12.2 (1.1)	
	Mexican American	9.2 (1.3)	6.7 (0.9)	
	Others	8.0 (1.5)	7.3 (1.2)	
Education				
	< High school	19.5 (1.6)	21.8 (1.3)	<0.05
	High school	25.0 (1.9)	29.5 (1.6)	
	College	55.5 (2.1)	48.7 (1.8)	
Income				
	<$20,000	17.9 (1.5)	23.8 (1.2)	<0.05
	$20,000-44,999	32.8 (2.0)	34.4 (1.9)	
	≥$45,000	49.3 (2.3)	41.8 (1.9)	
Smoking				
	Never	55.5 (2.3)	47.9 (1.6)	<0.05
	Current	21.3 (1.7)	20.8 (1.4)	
	Past	23.2 (2.1)	31.4 (1.3)	
Alcohol Intake				
	<3 drinks per day	63.2 (2.1)	68.6 (1.8)	<0.05
	≥3 drinks per day	36.8 (2.1)	31.4 (1.8)	
Recreational PA				
	Inactive	34.4 (2.0)	42.6 (1.5)	<0.05
	Somewhat Active	20.6 (1.5)	25.1 (1.5)	
	Active	45.0 (1.8)	32.3 (1.6)	
Transportation Physical Activity				
	Inactive	76.2 (2.1)	79.6 (1.2)	NS
	Somewhat Active	23.8 (2.1)	20.4 (1.2)	
	Active	-	-	
Household Physical Activity				
	Inactive	30.9 (2.5)	34.6 (1.7)	NS
	Somewhat Active	39.0 (2.1)	39.1 (1.4)	
	Active	30.2 (2.2)	26.3 (1.6)	
Total Physical Activity				
	Inactive	13.9 (1.2)	19.5 (1.3)	<0.05
	Somewhat Active	22.6 (1.9)	30.0 (1.4)	
	Active	63.5 (2.2)	50.5 (1.8)	
*Change in weight from 10 y ago*				
	Lost ≥30 kg	42.8 (2.4)	16.3 (1.2)	<0.05
	Lost <30 kg	7.5 (1.1)	13.5 (1.2)	
	Gained 0 - <10 kg	13.6 (1.4)	22.1 (1.1)	
	Gained 10 - <20 kg	19.7 (1.6)	26.4 (1.2)	
	Gained 20 - <30 kg	10.0 (1.1)	13.8 (1.1)	
	Gained 30 - <40 kg	4.1 (0.7)	4.6 (0.6)	
	Gained ≥40 kg	2.4 (0.7)	3.4 (0.5)	
*Change in weight since age 25 y*				
	Lost ≥30 kg	17.5 (2.2)	6.8 (0.8)	<0.05
	Lost <30 kg	6.3 (1.0)	4.1 (0.5)	
	Gained 0 - <10 kg	12.6 (1.1)	6.9 (0.6)	
	Gained 10 - <20 kg	19.6 (1.8)	20.5 (1.3)	
	Gained 20 - <30 kg	21.9 (1.9)	27.3 (1.1)	
	Gained 30 - <40 kg	13.8 (1.7)	18.7 (1.3)	
	Gained ≥40 kg	8.3 (1.2)	15.8 (1.2)	

Mean (SEM) for continuous variables and % (SE) for categorical variables. Body Mass Index (BMI: kg/m^2^). Physical Activity Adherence Categories: Inactive (No reported physical activity), Somewhat active (<500 MET min/week), Active (≥500 MET min/week). Metabolically Healthy Obese (≤2 metabolic syndrome components) as defined by the Harmonized interim statement. Metabolically Abnormal Obese (≥3 metabolic syndrome components). p < 0.05, two-sided; t-test or Chi-square, as appropriate. NS (not significant). Sum of weights: 29,150,635

**Fig. 1:**
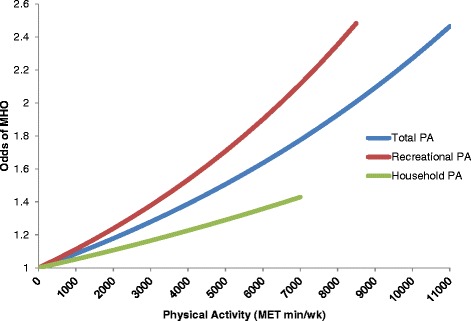
Odds of metabolically healthy obese phenotype by volume of total, recreational, and household physical activity domains. Energy expenditure (MET min/wk) calculations for PA domains are based on NHANES recommended MET values for recreational PA, 4.0 MET for moderate intensity activities, and 8.0 MET for vigorous intensity PA. Total PA is the sum of recreational-, household- and transportation-related PA

Figure 1 illustrates the relationship between PA volume and odds of MHO. Only total and recreational PA domains were significantly associated with MHO (β (SE) per 1000 MET min/wk: 0.078 (0.021) for total PA; and 0.102 (0.025) for recreational PA). No significant association was found for household PA, and due to insufficient data, we were unable to assess the relationship between MHO and transportation-related PA.

Table 2 contains the crude, age-adjusted and multivariable logistic regression models for the association between PA domains and MHO. In the crude analysis, those who accumulated over 500 MET min/week of total or recreational PA were 76 % and 72 % more likely to be MHO, respectively, as compared to their inactive peers. After adjusting for covariates, total PA, but not recreational remained associated with the MHO profile. Additionally adjusting for weight history lowered the odds of MHO by 3-10 % for 10 y weight history only (data not shown). Stratification by age revealed that meeting the PA guidelines was only associated with the MHO profile amongst those 45–59 years old (20–44 y: OR = 1.40, 0.85-2.30; 45–59 y: OR = 2.45, 1.10-5.46, and; ≥60 y: OR = 1.70, 0.65-4.43) (data not shown). However, age was not a moderator in the overall model (Wald χ^2^ for the interaction: 4.59; p = 0.10). Further analyses found no significant association between the number of MetS components and energy expenditure in PA domains (data not shown). In assessing the effect of PA volume and MHO phenotype, a significant association was only observed within groups who engaged in vigorous intensity recreational PA (Table 3). Here again, once adjusted for covariates, only 776.33-1382.82 MET min/week of vigorous intensity recreational PA remained significant (OR: 2.94 (95 % CI: 1.66, 5.23)).

**Table 2:** Odds of being metabolically healthy obese by PA domain

PA domain		Odds ratio (95 % CI)		
		OR_c_	OR_age_	OR_adj_
*Total PA*				
	Somewhat Active	1.05 (0.80, 1.39)	0.96 (0.71, 1.32)	1.18 (0.69, 2.01)
	Active	1.76 (1.36, 2.28)	1.48 (1.10, 1.99)	1.70 (1.19, 2.43)
*Recreational PA*				
	Somewhat Active	1.01 (0.77, 1.33)	0.92 (0.70, 1.21)	0.75 (0.51, 1.11)
	Active	1.72 (1.36, 2.18)	1.42 (1.11, 1.82)	1.26 (0.94, 1.68)
*Transportation PA*	Somewhat Active	1.22 (0.98, 1.52)	1.05 (0.83, 1.32)	1.15 (0.82, 1.63)
	Active	--	--	--
*Household PA*				
	Somewhat Active	1.12 (0.86, 1.45)	1.03 (0.77, 1.36)	0.92 (0.63, 1.33)
	Active	1.28 (0.99, 1.67)	1.28 (0.97, 1.69)	1.23 (0.89, 1.69)

Physical Activity Adherence Categories: Inactive (No reported physical activity, referent), somewhat active (<500 MET min/week), Active (≥500 MET min/week). Metabolically Healthy Obese (≤2 metabolic syndrome components). Odds Ratio (OR_c_) is crude odds ratio, OR_age_ is adjusted for age and OR_adj_ is adjusted for age, sex, education, income, ethnicity, smoking and alcohol. Note: Sample size for “Active” Transportation PA analysis was not adequate

**Table 3:** Odds of being metabolically healthy obese by recreational PA volume stratified by intensity

		Odds ratio (95 % CI)	
		OR_c_	OR_adj_
*Moderate Intensity (MET min/week)*			
	0	1.00	1.00
	>0-93.01	1.02 (0.64, 1.64)	0.75 (0.45, 1.24)
	>93.01-210.64	0.86 (0.57, 1.31)	0.74 (0.39, 1.41)
	>210.64-376.11	0.74 (0.49, 1.12)	0.49 (0.28, 0.85)
	>376.11-709.66	1.28 (0.88, 1.85)	0.90 (0.56, 1.42)
	>709.66	1.34 (1.02, 1.77)	1.11 (0.75, 1.62)
*Vigorous Intensity (MET min/week)*			
	0	1.00	1.00
	>0-195.02	1.85 (1.14, 3.03)	1.77 (0.92, 3.42)
	>195.02-430.64	1.70 (0.97, 2.97)	1.03 (0.52, 2.03)
	>430.64-776.33	1.63 (1.07, 2.48)	1.47 (0.86, 2.50)
	>776.33-1382.82	2.73 (1.60, 4.66)	2.94 (1.66, 5.23)
	>1382.82	1.88 (1.30, 2.72)	1.26 (0.85, 1.86)

PA category 0 min/week includes 0 minutes of activity per week and those without any reported recreational PA intensity data [10]. Metabolically Healthy Obese (≤2 metabolic syndrome components). Odds Ratio (OR_c_) is crude odds ratio and OR_adj_ is adjusted for age, sex, education, income, ethnicity, smoking and alcohol

**Fig. 2:**
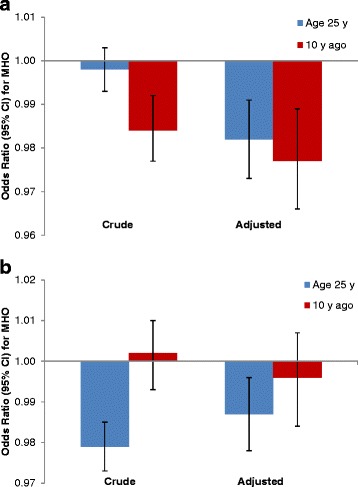
Relationship between weight history and metabolically healthy obese phenotype. Weight histories were modeled continuously in kg, while current MHO phenotype was dichotomous. Adjusted model controlled for age, sex, education, income, ethnicity, smoking and alcohol. (**a**) Weight (kg) at age 25 y and 10 y ago. (**b**) Change in weight (kg) from age 25 y and 10 y ago

Fewer MHO reported being overweight and obese 10 y ago and at age 25 y, and they gained approximately 5 kg less than the MAO since age 25 y (mean (95 % CI): 21.23 (20.08-22.52) vs. 26.11 (25.38-26.85 kg)). Moreover, change in weight (kg) from 10 years ago was not different between MHO vs. MAO ((12.05 (10.55-13.55) vs. (11.30 (10.55-12.04 kg)). A lower odds of MHO phenotype and weight at age 25 y, weight 10 y ago (Fig. 2a), and change in weight since age 25 y (Fig. 2b) was also observed, suggesting that weight history is significantly associated with MHO, even after adjustment for covariates. Additional adjustment for total PA did not materially change this relationship. Finally, for weight history, losing ≥30 kg within the past 10 y ago (vs. gaining <10 kg) was a consistent predictor of MHO, across all PA domains and PA guideline adherence (Table 4). However, adjusting for confounding variables attenuated these associations (Table 5). Conversely, gaining as little as 10 kg since age 25 y compared to gaining <10 kg lowered the odds of being MHO even in those adhering to the PA guidelines following adjustment for age, sex, ethnicity, income, education, alcohol intake and smoking history.

**Table 4:** Odds of being metabolically healthy obese by weight history, PA domains and PA adherence levels

		*Odds Ratio*_*MHO*_ *(95 % CI)*		
		PA adherence levels		
		Inactive	Somewhat active	Active
**Total PA**				
*Change in weight since age 25 y*				
	Lost ≥30 kg	1.20 (0.40, 3.64)	1.10 (0.41, 2.94)	1.62 (0.88, 2.98)
	Lost <30 kg	0.78 (0.17, 3.65)	0.70 (0.22, 2.25)	0.84 (0.41, 1.72)
	Gained 0 - <10 kg	1.00	1.00	1.00
	Gained 10 - <20 kg	0.52 (0.22, 1.24)	0.72 (0.33, 1.60)	0.44 (0.26, 0.74)
	Gained 20 - <30 kg	0.42 (0.17, 1.06)	0.44 (0.18, 1.06)	0.43 (0.25, 0.75)
	Gained 30 - <40 kg	0.27 (0.10, 0.73)	0.53 (0.22, 1.26)	0.40 (0.23, 0.69)
	Gained ≥40 kg	0.29 (0.12, 0.68)	0.43 (0.17, 1.12)	0.25 (0.14, 0.43)
*Change in weight from 10 y ago*				
	Lost ≥30 kg	5.16 (2.27, 11.71)	3.19 (1.60, 6.34)	4.66 (3.03, 7.15)
	Lost <30 kg	1.11 (0.37, 3.35)	0.68 (0.29, 1.58)	1.02 (0.58, 1.82)
	Gained 0 - <10 kg	1.00	1.00	1.00
	Gained 10 - <20 kg	2.45 (0.99, 6.08)	0.98 (0.50, 1.91)	1.12 (0.72, 1.72)
	Gained 20 - <30 kg	1.16 (0.61, 2.21)	1.58 (0.75, 3.35)	1.05 (0.62, 1.77)
	Gained 30 - <40 kg	2.07 (0.69, 6.20)	2.51 (0.97, 6.47)	1.05 (0.45, 2.42)
	Gained ≥40 kg	1.87 (0.52, 6.80)	1.25 (0.35, 4.42)	1.02 (0.41, 2.57)
**Recreational PA**				
*Change in weight since age 25 y*				
	Lost ≥30 kg	1.03 (0.48, 2.21)	1.45 (0.40, 5.21)	1.90 (0.90, 4.00)
	Lost <30 kg	0.69 (0.24, 1.98)	1.55 (0.49, 4.93)	0.79 (0.33, 1.85)
	Gained 0 - <10 kg	1.00	1.00	1.00
	Gained 10 - <20 kg	0.54 (0.29, 1.00)	1.04 (0.39, 2.76)	0.39 (0.19, 0.79)
	Gained 20 - <30 kg	0.44 (0.23, 0.85)	0.75 (0.26, 2.20)	0.37 (0.19, 0.73)
	Gained 30 - <40 kg	0.45 (0.22, 0.92)	0.73 (0.25, 2.08)	0.31 (0.14, 0.68)
	Gained ≥40 kg	0.26 (0.13, 0.50)	0.65 (0.19, 2.27)	0.25 (0.12, 0.50)
*Change in weight from 10 y ago*				
	Lost ≥30 kg	3.68 (2.06, 6.56)	4.20 (2.25, 7.87)	4.46 (2.93, 6.79)
	Lost <30 kg	1.00 (0.49, 2.04)	1.23 (0.43, 3.54)	0.66 (0.34, 1.29)
	Gained 0 - <10 kg	1.00	1.00	1.00
	Gained 10 - <20 kg	1.38 (0.72, 2.61)	1.50 (0.66, 3.42)	0.88 (0.57, 1.36)
	Gained 20 - <30 kg	1.61 (0.86, 3.02)	1.51 (0.75, 3.04)	0.67 (0.34, 1.32)
	Gained 30 - <40 kg	2.22 (0.97, 5.07)	1.46 (0.42, 5.07)	0.86 (0.29, 2.55)
	Gained ≥40 kg	1.04 (0.33, 3.32)	1.06 (0.26, 4.36)	1.20 (0.41, 3.53)
**Transportation PA**				
*Change in weight since age 25 y*				
	Lost ≥30 kg	1.74 (1.01, 3.00)	0.64 (0.30, 1.36)	-
	Lost <30 kg	1.07 (0.55, 2.07)	0.35 (0.10, 1.23)	-
	Gained 0 - <10 kg	1.00	1.00	-
	Gained 10 - <20 kg	0.60 (0.39, 0.94)	0.29 (0.13, 0.63)	-
	Gained 20 - <30 kg	0.54 (0.37, 0.78)	0.19 (0.08, 0.49)	-
	Gained 30 - <40 kg	0.52 (0.34, 0.79)	0.14 (0.06, 0.37)	-
	Gained ≥40 kg	0.35 (0.23, 0.55)	0.11 (0.04, 0.36)	-
*Change in weight from 10 y ago*				
	Lost ≥30 kg	4.40 (2.96, 6.55)	3.93 (2.33, 6.62)	-
	Lost <30 kg	0.82 (0.51, 1.31)	1.25 (0.57, 2.77)	-
	Gained 0 - <10 kg	1.00	1.00	-
	Gained 10 - <20 kg	1.20 (0.81, 1.76)	1.16 (0.57, 2.38)	-
	Gained 20 - <30 kg	1.25 (0.83, 1.88)	0.90 (0.44, 1.82)	-
	Gained 30 - <40 kg	1.70 (0.94, 3.08)	0.79 (0.25, 2.52)	-
	Gained ≥40 kg	1.14 (0.50, 2.56)	1.10 (0.34, 3.60)	-
**Household PA**				
*Change in weight since age 25 y*				
	Lost ≥30 kg	1.64 (0.73, 3.70)	1.37 (0.63, 3.01)	1.30 (0.49, 3.48)
	Lost <30 kg	1.07 (0.37, 3.14)	0.80 (0.33, 1.91)	0.72 (0.24, 2.12)
	Gained 0 - <10 kg	1.00	1.00	1.00
	Gained 10 - <20 kg	0.64 (0.30, 1.38)	0.42 (0.23, 0.79)	0.55 (0.27, 1.13)
	Gained 20 - <30 kg	0.37 (0.18, 0.77)	0.40 (0.20, 0.78)	0.56 (0.26, 1.19)
	Gained 30 - <40 kg	0.27 (0.13, 0.56)	0.49 (0.25, 0.96)	0.45 (0.20, 1.02)
	Gained ≥40 kg	0.32 (0.14, 0.70)	0.26 (0.11, 0.62)	0.30 (0.13, 0.69)
*Change in weight from 10 y ago*				
	Lost ≥30 kg	5.23 (3.00, 9.11)	3.53 (1.90, 6.56)	4.56 (2.31, 9.03)
	Lost <30 kg	0.60 (0.28, 1.26)	0.88 (0.46, 1.66)	1.63 (0.71, 3.75)
	Gained 0 - <10 kg	1.00	1.00	1.00
	Gained 10 - <20 kg	1.50 (0.86, 2.60)	1.12 (0.65, 1.91)	1.03 (0.54, 1.98)
	Gained 20 - <30 kg	1.02 (0.62, 1.67)	1.25 (0.64, 2.45)	1.33 (0.67, 2.62)
	Gained 30 - <40 kg	1.38 (0.58, 3.26)	1.55 (0.68, 3.56)	1.68 (0.51, 5.59)
	Gained ≥40 kg	2.86 (1.01, 8.15)	0.72 (0.19, 2.78)	0.83 (0.20, 3.52)

Physical Activity Categories: Inactive (No reported physical activity, referent), somewhat active (<500 MET min/week), Active (≥500 MET min/week). Metabolically Healthy Obese (≤2 metabolic syndrome components). Odds Ratio (OR_MHO_) is crude odds ratio. Note: Sample size for “Active” Transportation PA analysis was not adequate

**Table 5:** Odds of being metabolically healthy obese by weight history and PA adherence levels for total and recreational PA

		*Odds Ratio*_*age*_ *(95 % CI)*			*Odds Ratio*_*adj*_ *(95 % CI)*		
		PA adherence levels			PA adherence levels		
		Inactive	Somewhat active	Active	Inactive	Somewhat active	Active
**Total PA**							
*Change in weight since age 25 y*							
	Lost ≥30 kg	0.54 (0.17, 1.69)	0.56 (0.19, 1.67)	0.83 (0.44, 1.58)	0.46 (0.09, 2.31)	0.45 (0.11, 1.85)	0.70 (0.34, 1.45)
	Lost <30 kg	0.58 (0.11, 3.13)	0.56 (0.18, 1.74)	0.81 (0.41, 1.61)	0.52 (0.03, 7.81)	0.50 (0.12, 2.14)	0.45 (0.21, 0.97)
	Gained 0 - <10 kg	1.00	1.00	1.00	1.00	1.00	1.00
	Gained 10 - <20 kg	0.64 (0.25, 1.65)	0.75 (0.33, 1.68)	0.56 (0.33, 0.95)	1.72 (0.32, 9.24)	0.60 (0.20, 1.83)	0.49 (0.25, 0.93)
	Gained 20 - <30 kg	0.51 (0.19, 1.37)	0.59 (0.24, 1.42)	0.65 (0.37, 1.14)	0.99 (0.22, 4.35)	0.48 (0.15, 1.5)	0.48 (0.26, 0.88)
	Gained 30 - <40 kg	0.28 (0.10, 0.79)	0.66 (0.28, 1.58)	0.61 (0.33, 1.11)	0.37 (0.07, 1.89)	0.46 (0.14, 1.57)	0.52 (0.25, 1.09)
	Gained ≥40 kg	0.43 (0.17, 1.10)	0.52 (0.20, 1.36)	0.38 (0.21, 0.67)	0.6 (0.14, 2.52)	0.34 (0.09, 1.33)	0.24 (0.1, 0.55)
*Change in weight from 10 y ago*							
	Lost ≥30 kg	1.26 (0.47, 3.38)	1.5 (0.64, 3.55)	2.06 (1.21, 3.51)	1.09 (0.22, 5.37)	1.92 (0.55, 6.65)	1.66 (0.77, 3.54)
	Lost <30 kg	1.27 (0.41, 3.95)	0.68 (0.29, 1.60)	1.04 (0.58, 1.87)	0.8 (0.07, 9.36)	0.96 (0.28, 3.30)	0.73 (0.36, 1.49)
	Gained 0 - <10 kg	1.00	1.00	1.00	1.00	1.00	1.00
	Gained 10 - <20 kg	2.25 (0.92, 5.49)	0.85 (0.44, 1.65)	1.00 (0.66, 1.52)	3.1 (0.69, 13.88)	0.84 (0.4, 1.76)	0.97 (0.57, 1.63)
	Gained 20 - <30 kg	0.84 (0.43, 1.62)	1.39 (0.63, 3.08)	0.9 (0.53, 1.51)	0.64 (0.14, 2.85)	1.1 (0.43, 2.83)	0.80 (0.40, 1.61)
	Gained 30 - <40 kg	1.09 (0.37, 3.19)	1.93 (0.73, 5.15)	0.78 (0.32, 1.88)	0.19 (0.02, 2.14)	1.96 (0.48, 7.95)	0.57 (0.22, 1.49)
	Gained ≥40 kg	1.29 (0.33, 4.99)	0.92 (0.26, 3.3)	0.83 (0.34, 2.02)	0.65 (0.09, 4.77)	0.7 (0.11, 4.67)	0.83 (0.22, 3.15)
**Recreational PA**							
*Change in weight since age 25 y*							
	Lost ≥30 kg	0.53 (0.25, 1.12)	0.50 (0.12, 2.18)	1.11 (0.53, 2.34)	0.30 (0.11, 0.81)	0.95 (0.14, 6.49)	1.20 (0.56, 2.59)
	Lost <30 kg	0.6 (0.21, 1.76)	1.03 (0.32, 3.33)	0.78 (0.37, 1.66)	0.33 (0.08, 1.44)	1.22 (0.25, 6.02)	0.47 (0.18, 1.22)
	Gained 0 - <10 kg	1.00	1.00	1.00	1.00	1.00	1.00
	Gained 10 - <20 kg	0.65 (0.34, 1.25)	0.94 (0.36, 2.47)	0.49 (0.24, 0.99)	0.65 (0.27, 1.59)	1.50 (0.33, 6.84)	0.42 (0.19, 0.92)
	Gained 20 - <30 kg	0.62 (0.31, 1.24)	0.90 (0.32, 2.59)	0.55 (0.28, 1.09)	0.57 (0.24, 1.34)	1.30 (0.37, 4.54)	0.41 (0.21, 0.79)
	Gained 30 - <40 kg	0.58 (0.27, 1.22)	0.80 (0.28, 2.27)	0.48 (0.21, 1.11)	0.51 (0.18, 1.39)	1.06 (0.25, 4.39)	0.37 (0.14, 0.95)
	Gained ≥40 kg	0.37 (0.19, 0.74)	0.70 (0.20, 2.47)	0.39 (0.19, 0.81)	0.31 (0.11, 0.88)	0.53 (0.08, 3.37)	0.25 (0.09, 0.69)
*Change in weight from 10 y ago*							
	Lost ≥30 kg	1.28 (0.71, 2.30)	1.94 (0.87, 4.33)	2.15 (1.18, 3.91)	1.28 (0.54, 3.04)	2.38 (0.88, 6.41)	1.37 (0.58, 3.23)
	Lost <30 kg	1.04 (0.5, 2.16)	1.29 (0.45, 3.67)	0.67 (0.34, 1.31)	0.95 (0.36, 2.52)	1.56 (0.42, 5.78)	0.40 (0.16, 1.01)
	Gained 0 - <10 kg	1.00	1.00	1.00	1.00	1.00	1.00
	Gained 10 - <20 kg	1.29 (0.70, 2.39)	1.28 (0.57, 2.88)	0.79 (0.50, 1.22)	1.21 (0.58, 2.52)	2.06 (0.80, 5.27)	0.65 (0.38, 1.08)
	Gained 20 - <30 kg	1.36 (0.71, 2.61)	1.31 (0.66, 2.57)	0.57 (0.29, 1.11)	1.19 (0.43, 3.30)	1.51 (0.66, 3.46)	0.44 (0.18, 1.05)
	Gained 30 - <40 kg	1.47 (0.65, 3.32)	1.12 (0.30, 4.2)	0.65 (0.21, 1.96)	0.75 (0.24, 2.32)	1.51 (0.37, 6.15)	0.41 (0.11, 1.56)
	Gained ≥40 kg	0.82 (0.26, 2.59)	0.72 (0.17, 3.01)	0.97 (0.32, 2.90)	0.68 (0.16, 2.96)	0.19 (0.02, 1.84)	0.76 (0.15, 3.81)

Physical Activity Categories: Inactive (No reported physical activity, referent), somewhat active (<500 MET min/week), Active (≥500 MET min/week). Metabolically Healthy Obese (≤2 metabolic syndrome components). OR_age_ is adjusted for age and OR_adj_ is adjusted for age, sex, education, income, ethnicity, smoking and alcohol

## Discussion

The present study examined the relationship between energy expenditure in PA domains and weight history with MHO. Our results suggest that MHO are likely to have lower BMI, younger in age, be female, report higher volume of total and recreational PA, and vigorous intensity recreational PA. Once adjusted for potential confounders, only guideline adherence based on total PA and 776.33-1382.82 MET min/week of vigorous intensity recreational PA remained a significant predictor of the MHO profile. Further, age-stratified analysis revealed those in the 45–59 y age group for total PA domain benefited from meeting PA guidelines. Weight history analyses found that MHO gained less weight than MAO since age 25 y, and weight history, including change in weight, were significant predictors of the MHO profile.

Using the Harmonized Criteria for MetS, the prevalence of MHO in this study was 31.6 %, similar to other reports using the same criteria [7, 22]. In agreement with Wildman et al. [8] and Velho et al. [6], the MHO phenotype was associated with a younger age, female gender, and non-Hispanic black or Mexican American ethnicity. We found the prevalence of past smoking history was also lower in MHO, whereas others have reported that smoking status was not related to MHO [6] unless inflammation was included as a component of MAO [8]; however, research on the relationship between smoking, inflammation, and cardiometabolic risks is inconsistent [23]. These relationships are worthy of further study, given that weight gain is a commonly reported side effect of smoking cessation [24], and minimizing weight gain is important for maintaining the MHO phenotype [25].

### Association with physical activity

In the present study, odds of MHO were elevated amongst those who reported sufficient levels of total PA, effects that persisted after adjusting for covariates. These findings are consistent with earlier analyses from NHANES wherein Churilla and Fitzhugh [10] also found that leisure time and total PA offered protection from MetS, while domestic and transportation PA did not. By contrast, in the current analysis only total PA remained a significant predictor of MHO after adjustment for covariates. This may be partially explained by differences in population, PA adherence categorization, and covariates, as Churilla and Fitzhugh [10] additionally adjusted for family history of heart disease and diabetes. Taken together, our results suggest that total PA is more related to metabolic status amongst the obese population than recreational PA, whereas recreational PA may be more important in the general adult population [10]. However, only those engaged in higher levels of leisure time and total PA (i.e. >736.55 MET min/week and >1,261.18 MET min/week, respectively) were protected against MetS; and, subsequent analyses found only vigorous leisure time PA (>430.64 MET min/week) was protective against MetS [10]. In our study, we categorized the highest PA adherence level as ≥500 MET min/week to be consistent with the PA guidelines [26].

In other studies, engaging in light or moderate intensity activities has also been shown to protect individuals with obesity against MetS, but this effect is likely moderated by age [27]. Specifically, Camhi et al. [27] found that younger adults (19–44 y) who engaged in a greater volume of even light intensity activity were 2.7 times as likely to display the MHO phenotype as those who spent most of their day sitting; however, in adults aged 45–85 y, only moderate intensity PA was significantly associated with the MHO phenotype [27]. In our study, after stratifying by age, only adults aged 45–59 y who were active according to their total PA level were more likely to be MHO. In general, higher adherence levels of total or recreational PA were more strongly associated with the MHO phenotype. Indeed, an inverse association between leisure-time [28] and transportation-related PA [29, 30] and MetS has also been shown, and obese individuals who engage in transportation PA are also more likely to be metabolically healthy [27]. In the present study, no participants were sufficiently active based on transportation PA alone to be considered for separate analysis. Given that PA intensity has been shown to mediate the MetS-PA relationship [10], designation of all transportation PA as moderate intensity activity (4 MET) may have biased towards the null. Combined, these results suggest that intensity of activity may be an important feature of the PA-MHO relationship.

### Association with weight history

Consistent with the present analysis, previous studies that have found significant PA-MHO relationships [22, 31, 32] have also observed that MHO groups were younger and had lower average BMI than the MAO groups. For example, Camhi et al. [27] found that MHO young adults were four years younger and had an average BMI that was 2 kg/m^2^ lower than their MAO counterparts, whereas Wildman et al. [8] found that MHO were eight years younger than MAO. Taken together, these studies suggest that both age and weight gain are important predictors of the MHO phenotype. Further, in a study by Kuk and Ardern [33], the MAO group had a higher prevalence of overweight and obesity 10 y ago and at 25 y of age. As such, the MAO group had a higher weight and had experienced obesity for a longer duration, which may account in part to the greater degree of metabolic abnormalities [15]. Paradoxically, it has also been documented that an earlier age of onset of obesity is associated with a more favourable metabolic profile [32, 33]. Our finding of only modestly lower odds of MHO in those with higher weight 10 y ago and change in weight from age 25 y must therefore be interpreted with caution, given the potential for recall bias. To better understand the relationship between weight history, obesity and metabolic health, prospective studies simultaneously tracking objectively measured weight, adiposity and metabolic markers are therefore needed.

Finally, a recent meta-analysis by Kramer et al. [34] has challenged the existence of the metabolically “healthy” obese phenotype altogether, after finding that individuals with obesity are at an elevated risk of all-cause mortality, regardless of metabolic health. It is noteworthy, however, that this study was unable to account for duration of obesity or PA in their analysis. In the present analysis we evaluated the joint effect of weight history and PA adherence on the MHO phenotype and found that the strongest predictors of MHO were losing ≥30 kg from 10 y ago and not gaining more than 10 kg since age 25 y. Indeed, minimizing weight gain (rather than focussing on weight loss) in adulthood may be a more realistic target for clinical management of obesity-related comorbidities. Earlier analyses which have adjusted for PA found that only those who are obese and have significant obesity-related comorbidities are at an elevated risk for all-cause mortality [35]. The inclusion of PA in all future analyses may reconcile the competing notions of metabolic health and mortality in the obese.

### Limitations

First, these findings are cross-sectional and must be interpreted with caution, as causality cannot be inferred. Second, the use of self-reported PA and weight history are subject to recall and healthy responder bias, and a fixed MET value of 4 was applied for all transportation-related and household PA domains. Third, use of a different MHO definition would result in different prevalences, which in turn could alter the observed associations. Finally, dietary intake information was not accounted for, and warrants further consideration.

## Conclusions

Using a nationally representative sample of individuals living with obesity in the US, significant associations between the MHO phenotype and PA domains, PA guideline adherence, and weight history were observed. To better inform the treatment and management of obesity, future studies exploring the impact of obesity duration, dietary intake, and longer-term outcomes amongst MHO individuals are warranted.

## Abbreviations

MHO: Metabolically healthy obese
MAO: Metabolically abnormal obese
PA: Physical activity
NHANES: National Health and Nutritional Examination Survey
MET: Metabolic equivalent
US: United States
BMI: Body mass index
MetS: Metabolic syndrome
DHHS: Department of Health and Human Services
